# Infant and young child feeding practice among mothers-child pair in irrigated and non-irrigated areas of Dangila district, Northwest Ethiopia, 2020: a community based comparative cross-sectional study

**DOI:** 10.1186/s12887-024-04721-2

**Published:** 2024-05-09

**Authors:** Mulat Tirfie Bayih, Melesse Belayneh, Wasihun Mekonen

**Affiliations:** 1 Department of Public Health Nutrition and Dietetics, School of Public Health, College of Medicine and Health Science, Bahir Dar University, Bahir Dar, Ethiopia; 2 Department of Health System Management and Health Economics, School of Public Health, College of Medicine and Health Science, Bahir Dar University, Bahir Dar, Ethiopia

**Keywords:** IYCF practice, Irrigation, Ethiopia

## Abstract

**Background:**

The issue of Infant and Young Child Feeding practices was widespread; it was estimated that infants were not exclusively breastfed in the first six months of life. Complementary foods were frequently provided too soon or too late, and they were often nutritionally deficient. Even, there are nutrition-sensitive activities like irrigation schemes, evidence on infant and young child feeding practices between irrigated and non-irrigated areas is scarce or limited.

**Objective:**

To assess the prevalence of infant and young child feeding practices among 0–23 months of age children in irrigated and non-irrigated areas of Dangila District, North-west Ethiopia, 2020.

**Methods:**

A community based comparative cross-sectional study was conducted from Dec 1, 2020, to Jun 1, 2020. A stratified sampling technique was implemented to select 823 mothers with children age 0–23 months from irrigated (411) and non-irrigated (412) kebeles. Data were collected using structured questionnaires. Multivariable logistic regression was used to identify the associated factors of infant and young child feeding practice. Adjusted odds ratios with a 95% CI were used to determine the degree of association between the independent and outcome variables. A p-value < 0.05 was used as a cutoff point to declare statistically significant variables with the outcome variable.

**Results:**

Among 823 households visited, 802 respondents with a response rate of 97.8% in irrigated and 96.11% in non-irrigated areas gave complete responses. The overall prevalence of good IYCF practice was 62.5% (95% CI: 34.2, 41.3), and it shows a significant difference between irrigated (72.2%) and non-irrigated areas (52.8%). Moreover, the study identified that education primary and above (AOR = 1.889, 95% CI: 1.38, 2.648) knowledge above mean (AOR = 2.347, 95% CI: 1.555, 3.542), positive attitude (AOR = 1.716, 95% CI: 1.139, 2.587), PNC follow-up (AOR = 1.606, 95% CI: 1.154, 2.360), women’s decision-making power (AOR = 1.840, 95% CI: 1.226, 2.763), and multiple delivery (AOR = 0.352, 95% CI: 0.213, 0.583) were significant factors for IYCF among 0–23 month-old children.

**Conclusion and recommendations:**

The infant and young child feeding practice among 0–23 month-old children was better in an irrigated area than in a non-irrigated area. Promoting irrigation practices, empowering women, and strengthening postnatal care are recommended interventions to increase infant, young, and child feeding practices in the study area.

## Introduction

Infant and young child feeding (IYCF) is a set of recommendations to achieve optimal infant and young child feeding practice for 0-23months of age children [1]. The main component of IYCF practice includes: early initiation of breastfeeding (EIBF), exclusive breastfeeding (EBF) continued breastfeeding through age of 2yrs years and timely, adequate and safe complementary feeding (CF) and consumption of iron rich foods [2, 3].

IYCF practice is a cornerstone of care for child development mentally and growth physically, but it is often under estimated. Failure to proper infant and young child feeding practice is associated with increased risks of child health. Such as childhood morbidity, mortality, impaired motor, cognitive and behavioral development, slow physical growth, diminished immunity, reduced learning capacity and under-nutrition [4]. Poor nutrition leads to ill-health and ill-health contributes to further deterioration in nutritional status. 50 − 70% of the burden of diarrheal diseases, measles, malaria, and lower respiratory infections was attributable to malnutrition [5, 6].

World Health Organization (WHO) and United Nations International Children’s Fund (UNICEF) have developed the Global Strategy for IYCF practice. It recognizes appropriate infant and child feeding practices to improving nutritional status and decreasing infant mortality in all countries [3]. . Based on this, Ethiopian ministry of health (MOH) established the national nutrition program (NNP II) and the national guideline on adolescent, maternal, infant, and young child nutrition (AMIYCN) to promote optimal feeding practices. The improvement of infant and young child feeding has been the subject of numerous interventions including national nutrition policies, nutrition specific and sensitive intervention efforts. One of the methods for meeting the fundamental needs of the community is to increase agricultural productivity using irrigation systems to provide food security at the household level [4, 7–10]. 

The problem of IYCF practice is vast, it is estimated that 50% and 34.8% of infants are EIBF and EBF for the first 6 months of life respectively. Complementary foods are often introduced too early or too late and are often nutritionally inadequate and unsafe [11–13]. Which is surprise, only 18% of children received a minimum acceptable diet (MAD), 28% received diverse food groups and 55% were feed with minimum meal frequency (MMF) [14]. It is well recognized that the size of the problem is high in Africa, 51%, 37–40% and 40%, EBF, MMF and MDD respectively [5, 15, 16].

Different literatures, governmental and non-governmental reports argue that, IYCF practice is not well practiced globally and nationally [3, 17, 18]. Even these realities in Ethiopia, there were some studies were conducted to identify the prevalence and associated factors among children less than 2yrs [19–21]. However, most of those studies were conducted in urban area, which is difficult to generalize the findings to rural area and comparative cross-sectional study designs were not implemented. Important variables like house hold food security and attitude were not included. More over the evidence in irrigated and non-irrigated area is scarce or limited. This indicates that, it has a long way to go to fill these gaps. Therefore, the aim of this study is to compare infant and young child feeding practice among 0–23 months of age in irrigated and non-irrigated area.

## Methods and materials

### Study setting and period

The study was conducted in Dangila Woreda from Dec 1, 2020 to Jun 1, 2020. Dangila was found in Awi zone located 485 Km from the capital city Addis Ababa and 78 Km from regional city Bahir Dar. In the district there were Amhara and Agew elites with a total projected population of 156,169 in the year 2020. It is further divided into 6 sub clusters and 31 kebeles (Kebele is the lowest administrative unit in Ethiopia). Out of 31 kebeles, 10 kebeles were irrigation practiced and 21 kebeles were non-irrigation practiced. In Dangila district, there are 1 primary hospital (governmental), 6 Health Centers and 31 health posts. The district childbearing age groups were 34,825 of the total female population and under-five age groups were 21,145 among these under-two years were 7808 [22].

### Study design and populations

A Community based comparative cross-sectional study was conducted to assess the magnitude of IYCF practices of mothers who had infant and young children 0–23 months of age its associated factors in both irrigation users and non-irrigation user households. The source populations for the study were all mothers who had infant and young children 0–23 months of age residing in Dangila, Woreda and the study populations were all mothers who had infant and young children 0–23 months of age in the selected kebeles. All selected mothers who had infant and young children 0–23 months of age in each selected kebeles were the study unit and included. Mothers who had infant and young children 0–23 months of age those unable to communicate during data collection were excluded.

### Sample size determination

The required sample size of the study was determined by using double population proportion formula by considering the following assumption.95% confidence level,80% power, of the study, P1 and P2 the prevalence IYCF practice in irrigated area and non-irrigated area respectively. The two comparison groups population ratio 1:1, prevalence of infant and young child feeding practice (p2 = 43.4%) was taken from the previous studies done at North Achefer Woreda, Amhara, Ethiopia [21]. For irrigated area the prevalence of infant and young child feeding (p1 = 58.4%) was taken to detect 10% difference from non-irrigated area.

$$\varvec{n}1=\varvec{n}2=\frac{{({\varvec{z}}_{\frac{\varvec{\alpha }}{2}} \sqrt{2\stackrel{-}{\varvec{p}\varvec{q}}} +\varvec{z}\varvec{\beta }\sqrt{{\varvec{p}}_{1{\varvec{q}}_{1} +{\varvec{p}}_{2{\varvec{q}}_{2}}}})}^{2}}{{\varDelta }^{2}}$$

$$\text{W}\text{h}\text{e}\text{r}\text{e} = \stackrel{-}{\varvec{p}} = \frac{{\varvec{p}}_{1} +{\varvec{p}}_{2}}{2}$$

$$\stackrel{-}{\varvec{q}} = 1- \stackrel{-}{\varvec{p}}$$

$$\overrightarrow{p}$$

= 0.484,

$$\overrightarrow{q}$$

= 0.516, p1= 0.534, p2= 0.434

$$\text{q}1=1-\text{p}1=0.466$$

and

$$\text{q}2=1-\text{p}2=0.566$$,

$$\varDelta =\text{p}1-\text{p}2$$

=0.15

$$n1=\text{n}2=\frac{\left(1.96\right(\sqrt{2\left(0.484 \text{x} 0.516\right)} +0.84\sqrt{0.534 \text{x} 0.466+0.434 \text{x} 0.566} )2 }{(0.534-0.434)2}$$

Therefore, **n**_**1**_ **= n**_**2**_ **= 391**, the group sample was **782.** Finally, by taking 5% non-response rate the total sample size was **823.**

### Sampling technique and sampling procedure

A stratified random sampling method was implemented to identify irrigated and non-irrigated kebeles. After stratification, three kebeles from irrigated fields and six kebeles from non-irrigated fields were selected by using a simple random sampling technique (the lottery method). A proportional size allocation was used to determine the required sample size for each kebele. Finally, the sample was drawn from a list of infants and young children registered at the health post using a systematic simple random sampling technique.

### Variable and operational definition of terms

Infant and young child feeding practice was the dependent variable and Socio-demographic, Maternal and child health service, Household food security, Knowledge and attitude, Women decision power and social support were the independent variables.

#### Irrigated area

areas where a practice to river diversion, pumping, and small or large dam’s for agricultural cultivation during non-rainfall seasons in addition to rainfall seasons [23].

#### Appropriate IYCF practice/good

defined as early initiation of breast feeding within1hr after delivery, exclusive breast feeding to infant age less than 6 months, continue breast feeding 1yrs and above, timely introduction of solid, semi-solid and soft foods in 6–8 months of age, minimum dietary diversity, minimum meal frequency, minimum acceptable diet and consumption of Iron rich foods. A practice that was appropriate for a specific age group received a score of 1, and a practice that was inappropriate received a score of 0. If summed score of the indicators is equal to 4 or above ( above mean), it was considered as appropriate(good) IYCFP and If summed score of the indicators is equal to 3 or below ( below mean), it was considered as inappropriate(good) IYCFP [2, 24].

#### Early initiation of breastfeed

Proportion of children born in the last 23 months who were put to the breast within one hour of birth [25].

#### Exclusive breastfeeding (EBF)

means that an infant receives only breast milk from his or her mother or a wet-nurse, or expressed breast milk, and no other liquids or solids, not even water, with the exception of oral rehydration solution, drops or syrups consisting of vitamins, minerals supplements or medicines [13, 25].

#### Continued breastfeeding

continue breastfeeding for to 1yrs and above or more along with complementary feeding.

#### Introduction of complementary feeding

The process of introducing, solid, semi-solid or soft foods along with breast milk 6–8 months, when breast milk is no longer sufficient to meet the nutritional requirements of infants and young children [13].

#### Minimum dietary diversity

Proportion of children 6–23 months of age who receive foods from 4 or more food groups among the 7 food groups [25].

#### Minimum meal frequency

Proportion of breastfed and non-breastfed children 6–23 months of age who receive solid, semi-solid, or soft foods (but also including milk feeds for non-breastfed children. minimum frequency by age defined as: − 2 times for breastfed infants 6–9 months, 3 times for breastfed children 9–24 months and 4 times for non-breastfed children 6-24months. In this study the maximum value 4 was taken to compute meal frequency [25].

#### Minimum acceptable diet

Proportion of children 6–23 months of age who receive a mini- mum dietary diversity and minimum meal frequency (apart from breast milk) [25].

#### Consumption iron rich foods

Proportion of children 6–23 months of age who receive iron rich foods [25].

#### House hold food security

A state in which “all people at all times have both physical and economic access to sufficient food to meet their dietary needs for a productive and healthy life”. Measured by asking in the past four week’s household food status using yes or no questions. 0 = No (skip to Q—) 1 = Yes (1 = rarely (once or twice in the past four weeks, 2 = Sometimes (three to ten times in the past four weeks, 3 = Often (more than ten times in the past four weeks) Calculate the household food Insecurity access category for each household. 1 = Food Secure, 2 = Mildly Food Insecure Access, 3 = Moderately Food Insecure Access, 4 = Severely Food Insecure Access [26].

#### Women’s decision making

Participation of women’s from house hold decision making with their husband. In this study the measurement was by taking three question (No = 0, yes = 1) from Demography Health Information System (DHIS), among these questions the cumulative result = 3 women’s decision and 1,2 = no women’s decision making [27, 28].

#### Knowledgeable of IYCF

when the respondents correctly answer above mean of questions about IYCF knowledge [21].

#### Positive attitude about IYCF

when the respondents agree to favorable questions to appropriate IYCF [21].

### Data collection tool and method

The questionnaires were prepared after reviewing different literature developed for similar purposes by different authors. It was developed in English then translated into the local language (Amharic) and finally retranslated back to English to check its consistency. The questionnaires contained socio-demographic and economic, household food security, knowledge and attitude, maternal, child health service related factors, as well as information on women’s decision making power. Face-to-face interviews were used to collect data. The data collection was conducted in a calm and private environment to ensure confidentiality. The data collectors were four diploma nurses, with one health officer serving as the supervisor.

### Data quality control

In order to ensure the accuracy and consistency of the data collected, standardized data collection tools were developed in English and then translated to Amharic, the local language, for data collection and back to English for consistency. The developed questionnaire was pretested on 5% of the total sample size in other sites to evaluate its effectiveness. Prior to the actual data collection, two-day training was given to data collectors and supervisors on the selection procedure of study participants, the purpose of the study, and the steps to provide necessary information to participants. The supervisor and principal investigator monitored and ensured the completeness and quality of data daily. During data collection, the supervisor and principal investigators reviewed and checked the questionnaires for completeness and provided necessary feedback to the data collectors the following morning.

### Data processing and analysis

The collected data was processed using Epi-info data version 7 and exported to SPSS version 23 for further analysis. Descriptive statistics such as frequency, percentage, and mean were calculated for different variables. The chi-square test was used to examine the association between two populations. Bi-variable logistic regression analysis was performed to determine the crude association between each independent variable and the outcome variable, and crude odds ratios were calculated. Variables with a p-value < 0.25 were included in the multivariable logistic regression analysis models if they were associated with the dependent variable in the bi-variable analysis. The Hosmer-Lemeshow test was conducted to assess model fitness in the final model, and a p-value > 0.05 was considered to indicate a good fit. Odds ratios with a 95% confidence interval were estimated to evaluate the level of association, and a p-value less than 0.05 was considered significant.

### Ethical consideration

Letter of ethical approval was obtained from the Institutional Review Board (IRB) of Bahir Dar University, College of Medicine and Health Sciences (protocol number 00312/2020) and formal permission letter from Dangila administrative council and health office. Before collecting the data, verbal informed consent was taken from all subjects and their legal guardian(s) and the process of verbal informed consent was approved by the ethics committee (Institutional Review Board (IRB) of Bahir Dar University, College of Medicine and Health Sciences). Each study participants were informed about the purpose of the study and participation was voluntary without payment for their participation. Each study participants also were informed that the right to withdraw at any time during the interview. All gathered information were protected from its confidentiality, anonymity was explaining clearly to participant. Except the principal investigator information is not exposed third person.

## Results

### Socio demographic and economic characteristics of study participants

Among 823 households visited, 802 respondents with a response rate 97.4% participated in the study. Nearly half of the mothers (46.3% in irrigated and 48.48% in non-irrigated) found in the age class of 35 and above. Of those study participants 276 (68%) and 263 (66.4%) had 6-23months of age children in irrigated and in non-irrigated area respectively. The mean (± SD) age of children were 10.61(± 6.1) months and the mean age of mothers were 30.3 (± 6.2) years. Regarding to mother’s educational status, 213(52.5%) of mothers in irrigated and 182(46%) in non-irrigated areas had no formal education. Almost all the participants of this study 395(98%) in irrigated and 391(98.74%) in non-irrigated area were orthodox Christian followers. The Wealth index status of households 209(51.48%) and 134(33.8%) had higher asset of household economy among irrigated and non-irrigated area respectively (Table 1).

**Table 1:** Socio-economic and demographic characteristics of the respondents from irrigated and non-irrigated area of Dangila Woreda, north-west Ethiopia, 2021 (*n* = 802)

Characteristics		Kebele category code			Total		Chi-square( p. Value)
		Irrigated Area(*n* = 406)	Non-irrigated Area(*n* = 396)				
		Frequency (%)	Frequency (%)		Frequency (%)		
Age of mother							
16-25yrs		104 (25.6%)	98(24.7%)		202(25.2%)		0.050 (0.592)
25–34 yrs.		188 (46.31%)	197(49.7%)		385(48.0%)		
35 and above		114 (28.1%)	101(25.5%)		215(26.8%)		
Marital status							
Single		16(3.9%)	8(2.0%)		24(3.0%)		4.789(0.310)
Married		378(93.1%)	370(93.4%)		748(93.3%)		
Divorced		7(1.7%)	9(2.3%)		16(2.0%)		
Widowed		4(1.0%)	5(1.3%)		9(1.1%)		
Other		1(0.2%)	4(1.0%)		5(0.6%)		
Age of child							
0-less than 6 months		113(27.8%)	109(27.5%)		222(27.7%)		0.009(0.923)
6–23 months		293(72.2%)	287(72.5%)		580(72.3%)		
Sex of child							
Male		225(55.4%)	185(46.7%)		410(51.1%)		4.36(0.037)
Female		181(44.6%)	211(52.28%)		392(48.9%)		
Education of mother							
No formal education		213(52.46%)	182(46%)		395(49.3%)		11.095(0.001)
Primary and above		193(47.54%)	214(54%)		407(50.7%)		
Occupation of mother							
House wife		311(76.6%)	313(79%)		624(77.8%)		4.927(0.553)
Merchant		16(4.2%)	10(2.53%)		26(3.2%)		
Farmer		43(10.6%)	50(12.26%)		93(11.6%)		
Daily labor		27(6.7%)	18(4.5%)		45(5.6%)		
Other		9(2.2%)	5(1.4%)		14(1.7%)		
Religion							
Orthodox		395(97.3%)	391(98.74%)		786(98%)		2.369(0.306)
Muslim		8(2%)	3(0.7%)		11(1.4%)		
Other		3(0.7%)	2(0.5%)		5(0.6%)		
Educational status of husband							
No formal education		162(39.9%)	128(32.3%)		290(38.8%)		4.987(0.026)
Primary and above		244(60.1%)	268(67.7%)		512(63.8%)		
Occupation of husband							
Farmer		304(74.9%)	306(77.27%)		610(86.6%)		3.764(0.709)
Merchant		36(8.8%)	30(7.57%)		66(9.4%)		
Employed		27(7.2%)	27(7.2%)		54(7.2%)		
Other		18(4.43%)	10(2.52%)		28(4%)		
Total family size							
<=4		290(71.4%)	269(67.9%)		559(69.7%)		1.125(0.289)
=>4		116(28.57%)	127(32%)		243(30.3%)		
Wealth index							
Poor		8220 (17%)	125(31.6%)		207(25.8%)		27.132 (0.000)
Medium		115(28.33%)	137(34.6%)		252(31.4%)		
Higher/rich		209(51.48%)	134(33.8%)		343(42.8%)		

### Maternal and child health service utilization related characteristics of the respondents

Both in irrigated and non-irrigated area, more than nine out of ten (94.8%) and (93.4%) respectively had ANC follow up. Among these only 107(27.8%) in irrigated and 73(19.7%) in non-irrigated area had four and above ANC follow up and 329(85%) in irrigated and 312(84.3%) in non-irrigated area were counseled about IYCF practice during ANC follow up. Almost all 388(95.6%) in irrigated and 366(92.4%) in non-irrigated area were attending institutional delivery. Majority of the participants 276(68%) in irrigated and half 198(50%) in non-irrigated area had Post Natal Care follow up. (Table 2)

**Table 2:** Maternal and child related characteristics of the respondents from irrigated and non-irrigated area of Dangila Woreda, north-west Ethiopia, 2021 (*n* = 802)

Characteristics	Kebele category code		Total	Chi –square( p. Value)
	Irrigated Area(*n* = 406)	Non-irrigated Area(*n* = 396)		
	Frequency (%)	Frequency (%)	Frequency (%)	
History of ANC attendance				
No	21(5.2%)	26(6.6%)	47(5.9%)	0.705(0.401)
Yes	385(94.8%)	370(93.4%)	755(94.1%)	
Time of first ANC starting (*n* = 385)		(*n* = 370)		
1-4months of pregnancy	208(54%)	158(42.7%)	361(47%)	11.506(0.003)
5 and above months	177(46%)	212(57.3%)	394(52.2%)	
Number of ANC Follow up (*n* = 385)		(*n* = 370)		
Once	44(11.4%)	55(14.9%)	99(13.1%)	11.914(0.008)
Two times	108(28.1%)	144(38.9%)	252(33.4%)	
Three times	126(32.7%)	98(26.5%)	224(29.7%)	
Four and above	107(27.8%)	73(19.7%)	180(23%)	
IYCF counseling during pregnancy (*n* = 385)		(*n* = 370)		
No	56(14.5%)	58(15.7%)	114(15%)	1.925(0.165)
Yes	329(85.5%)	312(84.3%)	641(85%)	
Place of birth				
Health facility	388(95.6%)	366(92.4%)	756(94.4%)	5.702(0.017)
Home	18(4.4%)	30(7.6%)	48(5.6%)	
Birth attendant				
Health professional	388(95.6%)	366(92.4%)	756(94.4%)	3.034(0.082)
TBA	18(4.4%)	30 (7.6%)	48(5.6%)	
PNC follow up				
No	130(32%)	190(48%)	320(39.9%)	26.812(0.000)
Yes	276(68%)	206(52%)	474 (59.1%)	
IYCF counseling during PNC				
No	79(19.5%)	116(29.3%)	195(24.3%)	10.537(0.001)
Yes	327(80.5%)	280(70.7%)	607 (75.7%)	
Multiple delivery				
No	359(88.4%)	356(89.9%)	715(89.2%)	0.451(0.502)
Yes	47(11.6%)	40(10.1%)	87(10.8%)	
Birth order				
First	56(14%)	36(9%)	88(11.6%)	4.898(0.179)
Second and above	349(86%)	360(91%)	714(88.4%)	
Birth space				
2yrs and above	294(72.4%)	282(71.2%)	576(71.8%)	1.193(0.275)
Less than two yrs.	112(27.6%)	114(28.8%)	226(28.2%)	

### Knowledge, attitude, household food security status and women’s decision making characteristics

Based on knowledge score criteria responding above mean to knowledge assessment questions, 261(64.3%) in irrigated and 200(50.5%) in non-irrigated area were knowledgeable. More than half of the study participants 246(60.6%) in irrigated and 183(46.2%) in non-irrigated area had positive attitude toward infant and young child feeding practice. Nearly all of the participants (97.8%) in irrigated and (93.2%) in non-irrigated area were from food secured house hold. (Table 3)

**Table 3:** Knowledge, attitude, household food security status and women’s decision making of the respondents from irrigated and non-irrigated area of Dangila Woreda, north-west Ethiopia, 2021 (*n* = 802)

Characteristics	Kebele category code		Total	
	Irrigated Area(*n* = 406)	Non-irrigated Area(*n* = 396)		
	Frequency (%)	Frequency (%)	Frequency (%)	Chi-square (p-value)
Knowledge of respondents				
Knowledge below mean	145(35.7%)	196(49.5%)	341(42.5%)	15.577(0.000)
Knowledge above mean	261(64.3%)	200(50.5%)	461(57.7%)	
Attitude of respondents				
Negative attitude	160(39.4%)	213(53.8%)	373(46.5%)	16.660(0.000)
Positive attitude	246(60.6%)	183(46.2%)	429(53.5%)	
HH food security status				
Food insecure	9(2.2%)	27(6.8%)	36(4.5%)	9.900(0.002)
Food secure	397(97.8%)	369(93.2%)	766(95.5%)	
Women’s decision making				
No	291(71.7%)	316(79.8%)	607(75.7%)	7.188(0.007)
Yes	115(28.3%)	80(20.2%)	195(24.3%)	

### Prevalence of infant and young child feeding practices

The overall prevalence of good IYCF practice was 62.5% (95% CI: 34.2, 41.3) and it shows significant difference between irrigated (72.2%) and non-irrigated area. Three hundred five 75% and two hundred seventy 68.2% of mothers were initiated breast feeding within 1 h after delivery in the irrigated and the non-irrigated area respectively. Among breast feed mothers, 259(63.8%) in irrigated and 229(57.8%) in non-irrigated were practiced exclusively breast feed for the first six months. From the participants, 234(54.7%) in irrigated and 181(40.3%) in non-irrigated area were introduced complementary feeding at six months. Both the minimum dietary diversity 161(58.3%), minimum meal frequency 201(72.8%), and minimum acceptable diet was 124(44.9%) in irrigated area was higher than in non-irrigated area. (Table 4)

**Table 4:** Prevalence of infant and young child feeding of the respondents from irrigated and non-irrigated area of Dangila Woreda, north-west Ethiopia, 2021 (*n* = 802)

Characteristics	Kebele category code		Total	Chi-square (p.value)
	Irrigated Area(*n* = 406)	Non-irrigated Area(*n* = 396)		
	Frequency (%)	Frequency (%)	Frequency (%)	
Initiation of BF within 1 h after delivery				
No	101(25%)	126(31.8%)	227(28.3%)	4.760(0.029)
Yes	305(75%)	270(68.2%)	575(71.7%)	
Exclusive breast feeding				
No	147(36.2%)	167(42.2%)	314(39.2%)	2.994(0.084)
Yes	259(63.8%)	229(57.8%)	488(60.8%)	
Continued breast feeding to 1yrs				
No	18(4.4%)	26(6.5%)	44(5.5%)	2.712(0.258)
Yes	388(95.6%)	370(93.4%)	758(94.5%)	
Introduction of CF( n > = 6-23months = 276)		(n > = 6-23months = 263)		
No	125(45.3%)	157(59.7%)	282(52.3%)	11.424(0.001)
Yes	151(54.7%)	106(40.3%)	257(47.7%)	
Minimum dietary diversity(n > = 6-23mont = 276		(n >=6-23months = 263)		
No	115(41.7%)	195(74.1%)	310(57.5%)	50.146(0.000)
Yes	161(58.3%)	68(25.9%)	229(42.5%)	
Minimum meal frequency(n > = 6-23month = 276)		(n >=6-23months = 263)		
No	75(27.2%)	147(55.9%)	222(41.2%)	1.789(0.000)
Yes	201(72.8%)	116(44.1%)	317(57.8%)	
Minimum acceptable diet(n > = 6-23month = 276)		(n > = 6-23months = 263)		
No	152(55.1%)	200(76%)	352(67.3%)	25.337(0.000)
Yes	124(44.9%)	63(24%)	187(34.7%)	
Consumption of iron rich foods (n > = 6-23month = 276)		(n > = 6-23months = 263)		
No	73(26.4%)	130(49.4%)	203(37.7%)	52.836(0.000)
Yes	203(73.6%)	133(50.5%)	336(62.3%)	
Over all IYCF practice				
Poor	113(27.8%)	188(47.5%)	301(37.5%)	32.989(0.000)
Good	293(72.2%)	208(52.5%)	501(62.5%)	

### Factors associated with IYCF practice in irrigated and non-irrigated area

After checking all candidate variables in bi-variate analysis, variables with *p* < 0.25 were fitted to multivariable analysis. Irrigation status, women’s decision making power, education status of mothers, ANC follow up, place of birth, PNC follow up, multiple delivery, attitude and knowledge of mothers were significantly associated to infant and young child feeding practice. The odds of good IYCF practice in respondents who lived in irrigated area was 2.168 higher than who lived in non-irrigated area (AOR = 2.168, 95%CI: 1.529, 3.074). Mothers who had decision making power were 1.840 times more likely to had good IYCF practice than mothers who hadn’t decision making power (AOR = 1.840, 95% CI: 1.226, 2.763). The odds of good IYCF practice among mothers who had educational status of primary and above were1.889 times higher than mothers who had no formal education (AOR = 1.889, 95% CI: 1.38, 2.648). Mothers who had knowledge above mean were 2.347 times more likely to had good IYCF practice than mothers who had knowledge below mean (AOR = 2.347, 95% CI: 1.555, 3.542). The odds of good IYCF practice among mothers who had positive attitude were 1.716 times higher comparing to mothers who had negative attitude (AOR = 1.716, 95% CI: 1.139, 2.587). The odds of good IYCF practice among mothers who had PNC follow up were 1.606 higher than mothers who had not PNC follow up (AOR = 1.606, 95% CI: 1.154, 2.360). The odds of good IYCF practice among mothers who had multiple delivery were 0.352 times lesser than mothers who had not multiple delivery (AOR = 0.352, 95% CI: 0.213, 0.583). Table 5.

**Table 5:** Multivariable logistic regression analysis of factors associated with IYCF practice of the respondents from irrigated and non-irrigated area of Dangila Woreda, north-west Ethiopia, 2021 (*n* = 802)

Characteristics	IYCF practice (*n* = 802)				P value
	Good	Poor	COR(95%CI)	AOR(95%CI)	
Irrigation status					
Yes	293(58.5%)	113(37.5%)	2.344(1.748,3.142)	2.168(1.529,3.074)	0.000**
No	208(41.5%)	188(62.5%)	1		
Women’s decision making					
Yes	143(28.5%)	52(17.3%)	1.913(1.340, 2.731)	1.840(1.226, 2.763)	0.003*
No	358(71.5%)	249(82.7%)	1	1	
Education status of mothers					
Primary and above	308(61.5%)	149(49.5%)	1.628(1.220,2.173)	1.889(1.348,2.648)	0.000**
No formal education	193(38.5%)	152(50.5%)	1	1	
Family size					
Below 5	445(88.8%)	277(92%)	0.688(0.417,1.136)	0.650(0364,1.162)	0.146
Above 5	56(11.2%)	24(8%)	1	1	
ANC Follow up					
Yes	485(96.8%)	270(89.7%)	3.480(1.870, 6.479)	1.806(0.893, 3.655)	0.100
No	16(3.2%)	31(10.3%)	1	1	
Place of birth					
At home	18(3.6%)	27(9.0%)	1	1	
At health facility	483(96.4%)	274(91.0%)	0.378(1.430, 4.889)	0.507(0.246, 1.045)	0.066
Birth attendant					
Health professionals	479(95.6%)	276(91.7%)	1.972(1.091,3.564)	1.324(0.637,2.752)	0.452
Traditional birth attendance	22(4.4%)	25(8.3%)	1	1	
Multiple delivery					
Yes	35(7.0%)	52(17.3%)	0.36(0.228, 0.567)	0.352(0.213, 0.583)	0.000**
No	466(93.0%)	249(82.7%)	1	1	
PNC					
Yes	334(66.7%)	140(46.5%)	2.30(1.716, 3.083)	1.592(1.133, 2.234)	0.007*
No	167(33.3%)	161(53.5%)	1	1	
Wealth index					
Poor	137(27.3%)	70(23.3%)	1.399(0.977,2.004)	1.467(0.958,2.247)	0.078
Medium	164(32.7%)	88(29.2%)	1.332(0.952,1.865)	1.257(0.847,1.866)	0.256
Rich	200(39.9%)	143(47.5%)	1	1	
IYCF counseling during PNC					
Yes	400(79.8%)	207(68.8%)	1.798(1.297,2.495)	1.272(0.873,1.853)	0.210
No	101(20.2%)	94(31.2%)	1	1	
Attitude					
Positive	325(64.9%)	104(34.6%)	3.498(2.591, 4.721)	1.716(1.139, 2.587)	0.010*
Negative	176(35.1%)	197(65.4%)	1	1	
Knowledge					
Knowledge above mean	350(69.9%)	111(36.9%)	3.968(0.186, 0.341)	2.347(1.555, 3.542)	0.000**
Less knowledge below mean	151(30.1%)	190(63.1%)	1	1	
Food security status					
Secure	487(97.2%)	279(92.7%)	2.743(1.381,5.447)	1.044(0.465,2.346)	0.917
Insecure	14(2.8%)	22(7.3%)	1		

*P value < 0.05 **P value < = 0.001

## Discussions

The overall prevalence of good infant and young child feeding practice in this study was 62.5% (95% CI: 34.2, 41.3) and greater than the studies conducted Shashemene [24], North Achefer [21] and South Wollo Zone [29]. It shows statistically significant variation in irrigated and non-irrigated area among mothers who have 0–23 months of age children. The possible explanation for this significance variation might be due to irrigation can have a significant impact on smallholders’ livelihoods and food security. Because of improved productivity and changes in cropping patterns, irrigation can have a direct impact on food availability. When the productivity is increasing, economical assets and living status of the household becomes improved to feed their child. This an advantage to enhances timely introduction of complementary feeding, increases minimum dietary diversity, minimum mealy frequency and minimum acceptable diet results to improving IYCF practice in irrigated area than in non-irrigated area [30, 31].

The finding of this study revealed that two-third 75% in irrigated and 68.2% in non- irrigated area of respondent’s had early initiation of breast feeding within one hr. after delivery, which was higher than studies conducted in Kingdom of Saudi Arabia (43%) [32] and Nigeria 34.7% [33]. The deference might be low health facility delivery and skill birth attendant leads to missing the opportunity of early initiation of breast feeding by health professionals. It might be also health service performance and socio-cultural barriers. On the other hand, the finding of this study was consistent with EDHS survey analysis in Ethiopia (74.3%) [34] and study conducted in Assella town 70% [35]. It could be the focus and commitment of the government for child health and nutrition throughout the country is similar and dramatically increment of skill delivery. This might have the opportunity of initiating breast feeding within 1 h after delivery. More than half of the respondents (63.8%) in irrigated and 57.8% in non- irrigated area were exclusively breast feed for the first six months even without water. It was greater than studies conducted in Somaliland (20.47%) [36], Bishoftu (34.1%) [37] and East Gojam at Motta (50.1%) [38]. The discrepancy for this result might be due to socio-economic difference and cultural practice between study subjects in different part of Ethiopia. But lower than studies conducted in Assella town (86.3%) [35]. It might be residence, living in urban has an access to health service and media exposure to have information about breast feeding than those living in rural. continued breast feeding to 1yrs and above were (95.6%) in irrigated and 93.4% in non-irrigated area, which is higher than study conducted in Jima (75.6%) [39]. The probability of the difference might be, the majority of the participants in this study were housewives which could increase the likelihood of breastfeeding to their child, as it cost less when they have a poor economic status and they spend much of their time at home which increases the likelihood of continuing to breastfeed. Beyond this mothers in urban area might have workload, to turn their works mothers stop breast feeding early and use formula milk instead of breast milk. Urban mothers have better economical assets than those living in rural, based on this reality mothers in urban setting use breast milk substitution by commercially produced milk, cow milk and other commercially available foods due to easily accessible and ability of purchasing.

In this study timely introduction of complementary feeding at 6 months and above was found to be (54.7%) in irrigated and 40.3% in non-irrigated area, which was close to the study conducted in two Agro-ecological zone of Ethiopia (50.5%) [40]. On the contrary it was lower than studies conducted in India (72.7%) [41], Addis Ababa (81.1%) [42] and Jima (82.9%) [39]. This might be the difference between Indian and Ethiopian socio-economic level, cultural practice, accessibility of child foods and nutrition action intervention from ministry of health to health professionals, like health extension program implementation in Ethiopia. The difference between urban, rural and in deferent part of Ethiopia about awareness, economical status, health service accessibility and performance have its own influence on IYCF practice. Like ways minimum dietary diversity was (58.3%) in irrigated and 25.9% in non-irrigated area, it was greater than studies done at Northern India (29.6%) [43] and in Kenya (32–40%) [44], in Shashemene16.1% [24], in two Agro-ecological zone of Ethiopia (22.2%) [40], EDHS 2016 survey analysis (14.9%) [42] and Assella town (26.6%) [35]. This fact might be the study including irrigated area, enabling variety of food groups to be easily accessible and improve or growth of household economic status to feed diversified foods. Another reason might be, optimization of health extension program and community based neonatal care implementation was supported by Path finder from study area. Due to these facts the minimum dietary diversity becomes increased. Minimum meal frequency were (72.8%) in irrigated and 44.1% in non-irrigated area. This might be the level of awareness and media access about the frequency of complementary feeding. Minimum acceptable diet was (44.9%) in irrigated and 24% in non-irrigated, which was lower than as compared to studies conducted in India (45.8) [41] and Addis Ababa (65.1%) [42]. The minimum acceptable diet was greater than in India (19.5%) [41] and two Agro-ecological Zone of Ethiopia (12%) [40]. This discrepancy might be due irrigation scheme, socio-economic and cultural practice between country and study setting.

A significant association was observed between mothers’ participation on household decision making and good IYCF practice in both irrigated and non-irrigated area. Different studies support this study [45–47]. The prevalence of IYCF practice was significantly higher among those who had women’s decision making as compared to those who do not women’s decision making. The possible explanation might be when the mothers have decision making power on IYCF they can get autonomy to follow the appropriate child feeding practice and care. In addition to this, mothers who had participation on household decision making has freedom to visit health facilities for child health service with IYCF education [47, 48].

In this study PNC follow up was independently predictor to infant and young child feeding practice among 0-23months of age children. Mothers who had PNC follow up are receiving information to breast feeding, complementary feeding and diversified foods within cooking demonstration. Beside to this, health professionals may show practical demonstrations and role models for breast feeding and complementary feeding. Furthermore, it might be the strength of health extension worker implementation to maternal health service packages including postnatal service. This finding is supported by previous studies conducted in Assella [35], Shashemene [24].

Knowledge and attitude were significantly associated to infant and young child feeding practice. Mothers who were knowledgeable and mothers who had positive attitude were more likely practice infant and young child feeding practice. This might be those mothers having information and understanding about the issue of IYCF components can have a better chance of good IYCF practice. The same is true mothers who has positive inclination toward IYCF have a chance to increase IYCF practice. This result is supported by the previous studies conducted in Saudi Arabia [32], Uganda [49], in North west Ethiopia [48] and in Kenya [44].

Primarily the result of this study helps to improve IYCF feeding practice and child health. In addition to this, the finding of this research can also serve other researchers, educators, policy makers, governmental and nongovernmental organizations as a step point for initiation of activities and strengthening the utilization of available resources in order to decrease the child mortality ratio of our country which is much higher than the target.

### Strength and limitation of the study

The strength of this study was completing the on scheduled time bound and doing community based study during the era of covid-19 following prevention principles. The study has limitations. There might be a social desirability bias and a recall bias during answering of questions related to dietary practices and on house hold food security.

## Conclusion

The prevalence of infant and young child feeding practice among 0–23 months of age children was (62.5%) in the study area and had shown significant variation between irrigated and non-irrigated area. Women’s decision making, educational status, PNC follow up, attitude of mother/care giver and knowledge of mothers or care giver were identified as significant factors of infant and young child feeding practice among 0-23months of age children in the study area.

### Recommendation

#### For policy maker

Integrated Agricultural and Health Policies: Support policies that address both agricultural and health issues. Emphasize the need of policies that support irrigation infrastructure while simultaneously promoting health and nutrition program to ensure a comprehensive approach to child well-being.

#### For Dangila agricultural office

The district agricultural office gives great attention to extend irrigation schemes for non-irrigated area.

#### For the families and the community as whole

In order to improve infant and young child feeding practice the families and the communities would be egger on the issue and understanding the components of IYCF and practicing based health worker recommendations. The family also take/gain information about IYCF from nearby women’s development army, health extension worker and health worker. The families and communities also should attend cooking demonstration which is demonstrated by women’s development army and by health extension worker at health post to improve IYCF practice.

#### For rsesearcher

Consider conducting longitudinal studies to track the long-term effects of different feeding practices on child development and health outcomes. Explore the cultural and social factors influencing infant and young child feeding practices in both irrigated and non-irrigated areas through qualitative research methods.

## Data Availability

All the required data has been included in the manuscript.

## Abbreviations

AMIYCN: Adolescent maternal infant and young child nutrition
ANC: Antenatal care
CF: Complementary feeding
EBF: Exclusive breast feeding
EDHS: Ethiopian demographic and health survey
EIBF: Early initiation of breast feeding
EMDHS: Ethiopian mini-demographic and health survey
GDP: Gross domestic production
HSTP: Health sector transformation plan
IYCF: Infant and young child feeding
MAD: Minimum acceptable diet
MDD: Minimum dietary diversity
MDG: Millennium development goal
MMF: Minimum meal frequency
NICU: Neonatal intensive care unit
NNP: National nutrition program
PNC: Postnatal care
HH: Household
TBA: Traditional birth attendant
